# Plasma C-reactive protein is lower among marijuana using HIV-negative individuals but not among persons living with HIV

**DOI:** 10.1038/s41598-021-84352-0

**Published:** 2021-03-01

**Authors:** Ethan Morgan, Hannah Hudson, Richard D’Aquila, Brian Mustanski

**Affiliations:** 1 Infectious Disease Institute and College of Nursing, The Ohio State University, 1595 Neil Ave, Room 393, Columbus, OH 43210 USA; 2 Division of Infectious Diseases, Department of Medicine, Feinberg School of Medicine, Northwestern University, Chicago, IL USA; 3 Institute for Sexual and Gender Minority Health and Wellbeing, Northwestern University, Chicago, IL USA; 4 Department of Medical Social Sciences, Northwestern University, Chicago, IL USA

**Keywords:** HIV infections, Risk factors, Infectious diseases, Inflammation

## Abstract

The use of marijuana is highly prevalent among young men who have sex with men (YMSM). Past work has also shown that inflammation is elevated among YMSM, independent of HIV status. Here, we aim to examine the relationship between marijuana use and inflammation among this high-risk cohort, relative to use of other substances. Data were collected among YMSM aged 16–29 in Chicago. Multiplex cytokine and inflammatory biomarker assays were run on plasma from all persons living with HIV (PLWH) (n = 195) and a subset of HIV-negative participants (n = 489). Bivariate analyses and multivariable models assessed relationships between various substances and inflammatory biomarkers. Models were stratified by HIV status and adjusted for demographic characteristics. Most participants reported use of marijuana in the past 30 days (416, 60.8%). Mean blood C-reactive protein (CRP) levels were above the upper limit of normal (3.0 mg/L), indicative of increased risk for cardiovascular disease (mean CRP was 3.9 mg/L; SD = 8.5). In adjusted, stratified analyses, CRP was significantly lower among participants reporting frequent marijuana use (≥ 6 times per month), relative to those reporting never using marijuana, (β = − 0.38; 95% CI: − 0.73, − 0.03). However, this was entirely accounted for by an association among the HIV-negative participants and there was no significant association between marijuana use and blood CRP level among the PLWH. In summary, YMSM had markedly elevated marijuana use and blood CRP levels. Frequent marijuana use was associated with lower inflammation among only those not diagnosed with HIV. Further research is needed to explicate why there are differences between HIV-negative participants and PLWH and to leverage this information to characterize biological mechanisms by which marijuana decreases inflammation.

## Introduction

Developing a better understanding of how to reduce inflammation is clinically significant as consistently elevated levels of inflammation are associated with multiple aging-associated chronic diseases^1^. This is particularly significant in persons living with HIV (PLWH), among whom accelerated onset and increased severity of these “non-AIDS associated comorbidities” is an increasing disease burden^2, 3^. For example, C-reactive protein (CRP) is a biomarker for systemic inflammation. When its levels are consistently above the upper limit of normal (> 3 mg/L), the risk of cardiovascular disease increases about three-fold^4^. Despite the well established role that CRP-associated inflammation plays in chronic disease outcomes^5, 6^, and recent reports that it is elevated among young men who have sex with men and transgender women (YMSM/TGW) as a population^7, 8^, research in this area concerning health implications among YMSM/TGW is limited^7^.

Marijuana use is highly prevalent among young adults^9^ with use on the rise over the last decade^10^. Past research among sexual and gender minorities has demonstrated a higher prevalence of marijuana use compared to their heterosexual counterparts^11^ with some studies reporting rates use being nearly twice as high^12, 13^. A recent study among older adults on antiretroviral treatment for HIV infection found that heavy cannabis users, compared to non-users, had decreased frequencies of blood CD4 and CD8 T cells with markers of cell activation, including human leukocyte antigen (HLA)-DR and CD38+, although one of several monocyte subsets contributing to inflammation were found at increased frequency^14^.

Compounding any potential association between marijuana use and inflammation among YMSM/TGW is their increased likelihood of being diagnosed with human immunodeficiency virus (HIV) infection^15–18^. Interestingly, although HIV is known to increase systemic inflammation among infected individuals^19^, more recent work has found it to be elevated among YMSM/TGW as a population, regardless of HIV status^7, 8^. Additionally, the effectiveness of HIV preventative medication, pre-exposure prophylaxis or PrEP, has been demonstrated to be diminished among women with elevated levels of systemic cytokines (administered as a topical gel in this particular study)^18^. As such, it is timely to develop a better understanding how marijuana use affects systemic inflammation among YMSM/TGW who are, and are not, living with HIV, particularly in light of increasing marijuana legalization across the United States.

The use of substances other than marijuana has also been linked to elevated levels systemic inflammation^20, 21^. For example, among heterosexuals, the use of cocaine, methamphetamines, and opioids have each been demonstrated to have a pro-inflammatory effect^20, 21^. Further, the use of nicotine and heavy alcohol use have also been found to contribute to elevated levels of systemic inflammation, whether measured by CRP or pro-inflammatory cytokines^20, 22^. This body of research, however, has focused primarily on inflammation among the general adult population and does not directly address the higher rate of substance use among YMSM/TGW^23^, particularly as their patterns of substance use differ from the patterns observed among older MSM in terms of more frequent use of marijuana^24^.

Therefore, this paper is expanding upon past findings of high inflammation among this same cohort^8^, the RADAR study, to assesses the relationship between substance use and inflammation among YMSM/TGW with a specific focus on the use of marijuana. We hypothesized that, compared to those who report never using marijuana, YMSM/TGW who have used marijuana in the past six months will have significantly reduced systemic inflammation as measured by plasma CRP levels. To this end, we analyzed data from a large cohort of YMSM/TGW to: (1) assess systemic inflammation among marijuana using and non-using participants; (2) determine whether any potential association between marijuana use and inflammation differs according to HIV status; and (3) examine the relationship between inflammation and the use of various other substances.

## Methods

### Study design and recruitment

Data were collected as part of RADAR, an ongoing longitudinal cohort study of Chicago metropolitan area young sexual and gender minorities (YSGM) assigned a male sex at birth. The primary objective of this cohort study is to apply a multilevel perspective^25^ to a syndemic of health issues associated with HIV among YSGM^26^. Diverse methods for participant recruitment were used in order to achieve the multiple cohort, accelerated longitudinal design^27^. First, a subset of participants from two cohorts, Project Q2 and Crew 450, who were first recruited in 2007 and 2010, respectively, enrolled in RADAR. In 2015, a third cohort of YSGM was recruited. At the time of enrollment into their original respective cohorts, all participants were between 16 and 20 years of age, assigned male at birth, spoke English, and had a sexual encounter with a man in the previous year or identified as gay, bisexual or transgender. Lastly, cohort members were allowed to refer a maximum of three YSGM peers for enrollment into the study as long as they were between 16 and 29 years of age. All cohort members complete follow-up visits at six-month intervals and provided informed consent.

Informed consent was obtained from all study participants and all methods were carried out following relevant guidelines and regulations. For those participants 16–17 years of age, informed consent with an assessment of decisional capacity was obtained with an IRB-approved waiver of parental permission. After reaching the age of 18, an additional informed consent was obtained at the next study visit. All study procedures and protocol were approved by the Northwestern University Institutional Review Board (STU00087614).

### Measures

#### Demographics

Participants were asked to provide demographic information including age, race/ethnicity, current education level, and sexual orientation at each visit. Participants reporting a Hispanic/Latino ethnicity were coded as such, regardless of their racial identity. Measures used have been previously reported as part of this dataset^8, 28–30^.

#### Multiplex biomarker analyses

Biomarker measures have been previously described^8, 30^ and included CRP, Interleukin (IL)-1β, IL-6, IL-10, IL-15, macrophage inflammatory factor (MIP)-1a, MIP-1b, interferon (IFN)-γ, and tumor necrosis factor (TNF)-α. Each were tested for all PLWH (n = 195) and subset of HIV-negative participants (n = 489). No significant differences in demographic or risk behavior variables existed between the HIV-negative participants selected versus those not selected for these analyses. The analytic dataset includes 684 participants.

Multiplexed assays were conducted using the MESO QuickPlex SQ 120 electrochemiluminescence immunoassay platform (Meso Scale Discovery, MSD). This platform provides specificity along with a broad dynamic range. The MSD V-PLEX Plus Human CRP assay, for example, has a dynamic range of 0.001–49.6 mg/L and was used here. CRP results were validated using a second methodology, a particle enhanced immunoturbidimetric high-sensitivity (hs) CRP assay performed on a Roche/Hitachi cobas c 311 instrument (dynamic range: 0.15–20.0 mg/L) at North Shore Laboratory Services. Values between the two platforms were highly correlated (r = 0.99). Cytokines were also measured in participant plasmas using the MSD V-PLEX Custom Proinflammatory Panel 1 (human) kit**.**

#### Substance use behaviors

Marijuana use was operationalized and assessed in two ways. First, use was operationalized as a categorical variable^31^ based on self-reported frequency of use in the past thirty days: (1) never, (2) intermittent use, ≤ 5 times in the past 30 days, and (3) frequent, ≥ 6 times in the past 30 days. Second, marijuana use and associated problems were assessed using an eight-item screen instrument, the Cannabis Use Disorder Identification Test (CUDIT)^32, 33^. CUDIT scores ranged from 0–32 and were operationalized as a continuous variable with higher scores indicating more problematic use.

The use of various other substances (tobacco, cocaine or crack, heroin, methamphetamines, GHB, ketamine, poppers, inhalants, hallucinogens or psychedelics, ecstasy, stimulants, and prescription pain killers) was also assessed. To focus on more commonly used substances, only those substances whose self-reported prevalence was ≥ 4% in the sample were individually assessed (poppers, cocaine/crack, ecstasy, and psychedelics). Each of these assessments of individual substances were operationalized in two ways: (1) as a dichotomous variable indicating use or no use in the past six months; and (2) frequency of use categorized similar to marijuana use in the preceding paragraph. All other substances than marijuana were also combined into a single “other drug” use variable (methamphetamines, synthetic marijuana, ketamine, GHB, inhalants, and heroin) and operationalized as dichotomous variables indicating use or no use in the past six months.

In addition to self-reported use in the past six months, urine drug screening was conducted and results operationalized as urine drug screen positive or urine drug screen negative for any substance. To correspond to self-reported operationalization above, we individually examined urine drug screen results for marijuana/THC, cocaine, and methylenedioxymethamphetamine (MDMA/ecstasy). Other urine drug screen results were combined into a single variable (benzodiazepine, amphetamine, methamphetamine, and opiates). Metabolites were detected use the Ecstasy Drug Test (DMD-114) and Multi-Drug Screen Test (DOA-264) dip cards obtained from Innovacon, Inc. (San Diego, CA).

Alcohol use among participants was assessed using the Alcohol Use Disorders Identification Test (AUDIT)^34^ developed by the World Health Organization and was utilized as a continuous variable with higher scores indicating high levels of alcohol problems (possible range from 0–40). Current tobacco use was defined as ≥ 1 cigarette per day over the past 30 days. Sensitivity analyses were also run to combine all drug use except marijuana into a single binary variable indicating the use of any drug other than marijuana in the past six months or no drug use other than marijuana in the past six months.

#### HIV and STI testing

Fingerstick blood samples were collected as part of each participant’s visit every six months. Each participant’s HIV infection status was determined using the Alere Determine HIV1/2 Ab/Ag Combo 4^th^ generation point-of-care (POC) test. Those who tested positive on the POC HIV tests received confirmatory HIV antigen and antibody immunoassay testing following current CDC HIV testing guidelines^35^. Viral load testing was performed with the Abbott RealTime HIV-1 RNA PCR (sensitivity of 40 copies/mL). PLWH were categorized as having undetectable viral load if their laboratory results were < 50 copies/mL, detectable viral load was defined as ≥ 50 copies/mL^36, 37^. Regardless of participant’s self-reported STI history, we tested for both rectal gonorrhea and chlamydia via collection of rectal swabs.

### Statistical analyses

Chi-square and Student’s T-test statistics were used to determine whether or not demographic characteristics, marijuana use, and immunologic markers were independent from HIV status. All analyses included only data from the baseline visit of the RADAR study. Pearson’s correlation coefficients were used to test the relationship between key variables, CRP, and each of the immunologic cytokines; select key variables were dichotomous resulting in point-biserial correlation coefficients. Multivariable linear regression was then utilized to estimate the association between each of the marijuana use variables and CRP, adjusting for demographic characteristics. Secondary analyses were conducted to separately consider the relationship between frequency of use of other substances and inflammation. Sensitivity analyses were also conducted to: (1) assess whether positive STI test moderated the relationship between inflammation and marijuana; and (2) assess whether any interaction effect on inflammation existed between marijuana and tobacco use. All covariates identified as statistically significant at the *p* ≤ 0.05 level, using Wald test statistic, or known confounders were included in the multivariable regression models. All analyses were performed in Stata version 16.1.

## Results

Table 1 shows the demographic characteristics of the sample. The mean age of participants was 22.8 years (Standard Deviation [SD] = 4.8). Among the sample, 292 (42.7%) identified as black, 157 (23.0%) identified as white, 203 (29.7%) as Hispanic/Latinx, and 32 (4.7%) as a different or mixed race. Regarding sexual orientation, 483 (70.8%) identified as gay, 134 (20.0%) identified as bisexual, and 65 (9.5%) identified as a different sexual orientation. The majority of participants identified as cisgender male (624, 91.5%) while 47 (6.9%) identified as transgender woman and 11 (1.6%) identified as a different gender identity. 491 (72.0%) participants reported marijuana use in the past six months. Further broken down by frequency of use in the past 30 days, 159 (23.3%) reported intermittent use (≤ 5 times in the past 30 days),and 257 (37.7%) frequent use (≥ 6 times in the past 30 days). Other frequently used substances among this population included: poppers (108, 15.8%), cocaine/crack (100, 14.7%), ecstasy (75, 11.0%), and psychedelics (28, 4.1%). Urine drug screen results were positive for: marijuana use (297, 44.1%), cocaine/crack (38, 5.7%), and ecstasy (9, 1.3%). Among all participants, mean unadjusted CRP was 3.9 mg/L (SD = 8.5) and mean log-transformed CRP was 0.2 (SD = 1.6).

**Table 1:** Demographic characteristics, stratified by HIV status, RADAR, Chicago 2015–2017.

Variable	Total (N = 684)		HIV positive (n = 195)		HIV negative (n = 489)		p
	n/mean	%/SD	n/mean	%/SD	n/mean	%/SD	
**Age, mean (SD)**	22.8	4.8	25.0	4.3	22.0	4.7	< 0.001
**BMI, mean (SD)**	25.4	6.0	24.9	5.2	25.6	6.3	0.170
**Race/ethnicity, n (%)**							< 0.001
White	157	23.0	7	3.6	150	30.7	
Black	292	42.7	127	65.1	165	33.7	
Hispanic/Latinx	203	29.7	44	22.6	159	32.5	
Other	32	4.7	17	8.7	15	3.1	
**Education, n(%)**							0.259
< High school	100	14.7	23	11.8	77	15.8	
High school/GED	165	24.2	56	28.7	109	22.4	
Some college	334	49.0	92	47.2	242	50.0	
≥ Bachelor’s	83	12.2	24	12.3	59	12.1	
**Sexual orientation, n (%)**							0.003
Gay	483	70.8	155	79.5	328	67.4	
Bisexual	134	20.0	23	11.8	111	22.8	
Other	65	9.5	17	8.7	48	9.9	
**Gender identity, n(%)**							0.007
Male	624	91.5	172	88.2	452	92.8	
Transgender woman	47	6.9	22	11.3	25	5.2	
Other	11	1.6	1	0.5	10	2.1	
**Marijuana use frequency, n (%)**							0.002
Never	266	39.0	62	31.8	204	41.9	
Intermittent	159	23.3	39	20.0	120	24.6	
Frequent	257	37.7	94	48.2	163	33.5	
**Positive rectal STI test, n(%)**							
Gonorrhea	68	10.2	40	21.4	28	5.8	< 0.001
Chlamydia	83	12.4	40	21.3	43	8.9	< 0.001
**Drug and alcohol use**							
*Self-reported substance use*^1^							
Marijuana	491	72.0	144	73.9	347	71.3	0.496
Poppers	108	15.8	32	16.4	76	15.6	0.795
Cocaine/crack	100	14.7	37	19.0	63	12.9	0.044
Ecstasy	75	11.0	25	12.8	50	10.3	0.335
Psychedelics	28	4.1	1	0.5	27	5.5	0.003
Other drug use^2^	46	6.7	19	9.7	27	5.5	0.047
*Urine drug screen positive*^*3*^							
Marijuana	297	44.1	127	65.8	170	35.3	< 0.001
Cocaine/crack	38	5.7	23	11.9	15	3.1	< 0.001
Ecstasy/MDMA	9	1.3	6	3.1	3	0.6	0.011
Other drug use^4^	51	7.5	28	14.4	23	4.7	< 0.001
AUDIT^4^, mean (SD)	6.0	5.7	5.8	6.2	6.1	5.5	0.437
CUDIT^4^, mean (SD)	6.4	6.4	7.3	6.5	6.1	6.3	0.017
Tobacco use^5^, n(%)	292	42.8	106	54.4	186	38.2	< 0.001
**Biologic markers**							
Unadjusted CRP (mg/L), mean (SD)	3.9	8.5	5.7	9.8	3.3	7.9	< 0.001
Log-transformed CRP, mean (SD)	0.2	1.6	0.8	1.5	0.02	1.6	< 0.001
Detectable HIV viral load^6^	—	—	102	76.1	—	—	—

BMI = body mass index; STI = sexually transmitted infection; CRP = C-reactive protein.

^1^In the past six months.

^2^Other drug use includes: methamphetamines, synthetic marijuana, ketamine, GHB, inhalants, heroin.

^3^Note: urine drug screens unavailable for poppers and psychedelics; other urine drug screen includes: benzodiazepines, amphetamines, methamphetamines, opiates.

^4^Based on the Alcohol Use Disorders Identification Test (AUDIT) and Cannabis Use Disorders Identification Test (CUDIT) scoring methods; higher score indicates higher risk alcohol use.

^5^In the past 30 days.

^6^Detectable viral load defined as ≥ 50 copies/mL; viral load data available on 134 participants only.

Table 2 depicts the correlation between different biomarkers of inflammation and the use of various substances among PLWH. C-reactive protein was significantly correlated with MIP-1b (r = 0.37, p < 0.001), IFN-γ (r = 0.46, p < 0.001), IL-10 (r = 0.49, p < 0.001), IL-6 (r = 0.59, p < 0.001), and TNF-α (r = 0.44, p < 0.001). Other significant correlations among PLWH existed between self-reported psychedelic drug use and CRP (r = 0.22, p = 0.002), MIP-1b (r = 0.50, p < 0.001), IL-10 (r = 0.49, p < 0.001), and TNF-α (r = 0.15, p = 0.036); urine screen cocaine/crack and TNF-α (r = 0.18, p = 0.011); urine screen ecstasy/MDMA and MIP-1b (r = 0.14, p = 0.044); and urine screen for other drugs and TNF-α (r = 0.17, p = 0.020). Other significant correlations existed between several other risk behaviors and biomarkers, refer to Table 2 for all other correlations and Supplemental Table 1 for all p-values.

**Table 2:** Correlations between inflammatory cytokines, CRP, and various substances among persons living with HIV, RADAR, Chicago, 2015–2017.

Variable	CRP	IL-15	MIP-1a	MIP-1b	IFN-γ	IL-10	IL-1β	IL-6	TNF-α
**BMI**	0.14	− 0.06	0.09	− 0.03	0.002	− 0.05	− 0.03	0.02	− 0.01
**Tobacco use**^**1**^	0.07	− 0.01	− 0.03	0.01	− 0.05	− 0.06	0.09	0.05	0.005
**Self-reported substance use**^**2**^									
Marijuana	0.02	0.06	− 0.04	− 0.04	− 0.02	− 0.02	0.01	− 0.11	− 0.04
Poppers	0.01	0.08	− 0.08	0.01	0.09	− 0.06	0.01	0.0002	0.03
Cocaine/crack	− 0.04	0.08	− 0.001	− 0.03	− 0.06	− 0.07	0.10	− 0.02	0.05
Ecstasy	0.04	− 0.02	0.07	0.09	− 0.03	0.02	0.06	− 0.03	0.11
Psychedelics	0.22**	− 0.05	− 0.002	0.50***	− 0.02	0.49***	0.01	0.11	0.15*
Other drug use^3^	0.02	0.02	− 0.06	0.03	− 0.001	− 0.06	0.13	0.03	0.02
**Urine drug screen positive**^**4**^									
Marijuana	0.04	0.12	− 0.02	− 0.04	0.03	0.07	0.11	0.03	0.03
Cocaine/crack	− 0.03	0.02	0.005	0.03	− 0.02	0.07	0.01	0.13	0.18*
Ecstasy/MDMA	0.13	− 0.10	− 0.03	0.14*	− 0.06	0.17	0.11	0.06	0.05
Other drug use^5^	0.06	− 0.08	− 0.07	− 0.02	0.02	− 0.05	0.04	0.04	0.17*
**AUDIT score**^**6**^	− 0.05	0.12	− 0.05	− 0.06	− 0.04	− 0.04	− 0.02	− 0.02	− 0.04
**CUDIT score**^**6**^	0.03	0.002	− 0.002	− 0.04	0.06	− 0.02	0.18*	− 0.08	0.04
**Rectal gonorrhea**	0.08	− 0.07	0.03	0.07	− 0.003	0.20**	− 0.05	0.09	0.22**
**Rectal chlamydia**	0.10	0.08	0.14	0.02	0.12	0.12	− 0.03	0.16*	0.20**
**Undetectable HIV viral load**	0.03	0.06	0.03	− 0.07	0.21*	0.14	− 0.07	0.12	0.15
**CRP**	—	− 0.08	0.09	0.37***	0.46***	0.49***	0.12	0.59***	0.44***

*p < 0.05; **p < 0.01; ***p < 0.001.

^1^In the past 30 days.

^2^In the past six months, only those self-reported use ≥ 4%.

^3^Substance with < 4% self-reported use in the sample: methamphetamines, synthetic marijuana, ketamine, GHB, inhalants, heroin.

^4^Note: urine drug screens unavailable for poppers and psychedelics.

^5^Other urine drug screens include: benzodiazepines, amphetamines, methamphetamines, opiates.

^6^Based on the Alcohol Use Disorders Identification Test (AUDIT) and Cannabis Use Disorders Identification Test (CUDIT) scoring methods; higher score indicates higher risk alcohol use.

Table 3 depicts the correlation between different biomarkers of inflammation and the use of various substances among HIV-negative participants. C-reactive protein was significantly correlated with IL-15 (r = 0.16; p = 0.003) MIP-1b (r = 0.10, p = 0.022), IFN-γ (r = 0.27, p < 0.001), IL-10 (r = 0.16, p < 0.001), IL-6 (r = 0.50, p < 0.001), and TNF-α (r = 0.20, p < 0.001).

**Table 3:** Correlations between inflammatory cytokines, CRP, and various substances among HIV-negative participants, RADAR, Chicago, 2015–2017.

Variable	CRP	IL-15	MIP-1a	MIP-1b	IFN-γ	IL-10	IL-1β	IL-6	TNF-α
**BMI**	0.18***	0.03	0.001	0.23***	− 0.08	− 0.03	0.06	0.30***	0.12*
**Tobacco use**^**1**^	− 0.05	0.03	0.01	− 0.01	− 0.02	0.09	− 0.01	− 0.03	0.05
**Self-reported substance use**^**2**^									
Marijuana	− 0.13**	0.02	− 0.001	− 0.02	− 0.10*	0.004	0.04	− 0.13**	0.01
Poppers	− 0.01	− 0.06	0.01	− 0.01	− 0.01	− 0.01	0.06	0.02	0.07
Cocaine/crack	− 0.01	− 0.01	0.10*	− 0.05	− 0.02	0.02	− 0.001	− 0.05	0.11*
Ecstasy	− 0.01	0.05	0.01	− 0.04	− 0.03	− 0.02	0.02	− 0.05	0.04
Psychedelics	− 0.05	− 0.02	− 0.03	− 0.003	0.03	− 0.02	− 0.03	− 0.01	0.09
Other drug use^3^	− 0.02	0.06	− 0.01	-0.05	0.07	0.12**	0.04	− 0.02	0.12**
**Urine drug screen positive**^**4**^									
Marijuana	− 0.08	0.14**	0.06	0.11*	− 0.03	0.09*	0.05	− 0.004	0.009
Cocaine/crack	− 0.02	-0.003	− 0.02	0.02	− 0.02	0.05	0.10*	− 0.02	0.08
Ecstasy/MDMA	− 0.01	0.03	0.30***	0.05	− 0.01	0.02	− 0.02	0.01	− 0.02
Other drug use^5^	0.05	0.03	0.01	0.02	0.02	0.10*	0.08	0.01	0.02
**AUDIT score**^**6**^	− 0.07	-0.11*	0.03	-0.06	− 0.06	− 0.006	0.20***	− 0.06	0.05
**CUDIT score**^**6**^	− 0.11*	0.004	0.02	0.09	− 0.05	0.12**	− 0.02	− 0.07	0.07
**Rectal gonorrhea**	0.004	0.05	− 0.006	0.13**	0.005	0.15**	0.02	0.06	0.11*
**Rectal chlamydia**	− 0.03	0.07	0.04	0.10*	0.02	0.08	0.04	0.004	0.11*
**Undetectable HIV viral load**	−	−	–	−	–	−	−	−	−
**CRP**	−	0.16***	− 0.01	0.10*	0.27***	0.16***	0.01	0.50***	0.20***

*p < 0.05; **p < 0.01; ***p < 0.001.

^1^In the past 30 days.

^2^In the past six months, only those self-reported use ≥ 4%.

^3^Substance with < 4% self-reported use in the sample: methamphetamines, synthetic marijuana, ketamine, GHB, inhalants, heroin.

^4^Note: urine drug screens unavailable for poppers and psychedelics.

^5^Other urine drug screens include: benzodiazepines, amphetamines, methamphetamines, opiates.

^6^Based on the Alcohol Use Disorders Identification Test (AUDIT) and Cannabis Use Disorders Identification Test (CUDIT) scoring methods; higher score indicates higher risk alcohol use.

Self-reported marijuana use was significantly correlated with CRP (r = − 0.13, p = 0.003), IFN-γ (r = -− 0.10, p = 0.030), and IL-6 (r = -− 0.13, p = 0.004); urine screen positive for marijuana use was correlated with IL-15 (r = 0.14, p = 0.003), MIP-1b (r = 0.11, p = 0.016), and IL-10 (r = 0.09, p = 0.047). Self-reported cocaine/crack use was correlated with MIP-1a (r = 0.10 p = 0.026) and TNF-α (r = 0.11, p = 0.016); urine screen positive for cocaine/crack was associated with IL-1β (r = 0.10, p = 0.024) . Urine screen positive for ecstasy was significantly correlated with MIP-1a (r = 0.30, p < 0.001). Other significant correlations existed between several other risk behaviors and biomarkers, refer to Table 3 for correlation values and Supplemental Table 2 for p-values.

Results of the multivariable regression analyses examining the relationship between marijuana use and CRP are presented in Table 4. Results are stratified based on HIV status. Among HIV-negative participants, a significant association was observed between log CRP and frequency of marijuana use. In this relationship, compared to those who never used marijuana, frequent marijuana users had significantly lower CRP (β = − 0.38; 95% Confidence Interval [CI]: − 0.73, − 0.03). No significant relationship was observed between marijuana use and CRP among PLWH. Nor was any significant relationship observed between dichotomous use of any other substance and CRP among either HIV-negative or PLWH. Among PLWH, detectable viral load (≥ 50 copies/mL), compared to undetectable viral load (< 50 copies/mL), was significantly associated with log CRP (β = 0.69; 95% CI: 0.04, 1.34). Sensitivity analyses observed no moderation effect of STIs on the relationship between inflammation and marijuana use nor was there any significant interaction effect between marijuana and tobacco use. No significant relationship with CRP was observed in additional analyses separately examining frequency of use of self-reported poppers, cocaine/crack, ecstasy, psychedelics, and other drugs.

**Table 4:** Adjusted multivariable linear regression results examining the association between marijuana use and log CRP among HIV-negative participants and persons living with HIV (PLWH), RADAR, Chicago, 2015–2017.

Characteristic	HIV-negative		PLWH	
	Beta	95% CI	Beta	95% CI
Age	0.06**	0.02, 0.10	− 0.04	− 0.13, 0.05
BMI	0.08***	0.06, 0.10	0.08***	0.03, 0.13
**Race/ethnicity**				
White	Ref	–	Ref	–
Black/AA	0.002	− 0.39, 0.40	0.19	− 2.73, 3.11
Latinx	0.16	− 0.22, 0.53	0.93	− 2.01, 3.87
Multi-Racial/other	0.05	− 0.78, 0.89	0.25	− 2.81, 3.31
**Education**				
< High school	Ref	–	Ref	–
High school/GED	0.43	− 0.06, 0.92	0.35	− 0.50, 1.21
Some college	0.30	− 0.14, 0.74	0.32	− 0.48, 1.11
≥ Bachelor’s	− 0.10	− 0.79, 0.59	− 0.67	− 1.78, 0.44
**Sexual orientation**				
Gay	Ref	–	Ref	–
Bisexual	0.09	− 0.27, 0.45	− 0.13	− 0.96, 0.69
Other	− 0.42	− 0.91, 0.06	− 0.05	− 1.03, 0.92
**Marijuana use**				
Never	Ref	–	Ref	–
Intermittent	− 0.23	− 0.60, 0.14	0.29	− 0.46, 1.05
Frequent	− 0.38*	− 0.73, -0.03	0.46	− 0.18, 1.10
**Any other drug use**	0.24	− 0.10, 0.57	0.04	− 0.59, 0.66
**Detectable viral load**^**1**^	–	–	0.69*	0.04, 1.34

*p < 0.05; **p < 0.01; ***p < 0.001.

^1^Defined as ≥ 50 copies/mL.

## Discussion

Among a diverse population of young men who have sex with men and transgender women in Chicago, we assessed the relationship between the use of various substances and inflammation, with a particular focus on a hypothesis that marijuana use decreases inflammation. This hypothesis was partially supported by the results presented here. We observed that HIV-negative individuals who were also frequent users of marijuana (≥ 6 times per month), but not intermittent users (≤ 5 times per month), had significantly lower C-reactive protein compared to non-users. However, no significant relationship between marijuana use and CRP was observed among the YMSM/TGW PLWH. Additionally, no association was observed between frequency of use of other substances and CRP among either PLWH or HIV-negative participants. Correlations between other risk and behavioral variables and inflammation-associated cytokines varied widely. Further research is needed to assess the longitudinal effects of drug use on inflammation among this population, particularly as it appears to differ based on HIV diagnosis.

Past research has observed an association between cannabinoids and a reduction in levels of inflammation^38–40^. This earlier work, however, was conducted among primarily either laboratory animals or heterosexual and older populations. In this novel work among YMSM/TGW that extends to a much younger age range, we observed findings consistent with the earlier reports, but only among our HIV-negative participants. The effects of marijuana on inflammation are hypothesized to be due primarily to its ability to inhibit cytokine production and suppress T cell function including cell-mediated immunity^41^. Research on cannabinoids found in marijuana (e.g. tetrahydrocannabinol [THC], cannabidiol) have demonstrated meaningful anti-inflammatory effects due to, among other things, their ability to specifically suppress cytokine production and T cell activation^14, 42^. Cannabidiol is of particular interest as research has demonstrated its ability to reduce inflammation while avoiding the psychoactive side effects associated with marijuana use^43^. Further research has also found that cannabidiol use may improve inflammatory chronic diseases^43^ and may slow the effects of coronary artery disease^44^. Findings such as these may be key among the high-risk population studied here, given past work which has demonstrated that YMSM/TGW are at an increased risk of cardiovascular disease from a young age^8, 30^. Future research should aim to develop a better understanding of the longitudinal relationship between marijuana use and inflammation among this high risk population, particularly in terms of its ability to reduce subsequent mid- and late-life risk of chronic diseases.

In contrast to our findings among HIV-negative individuals, we observed no significant relationship between marijuana use and CRP among those diagnosed with HIV. Among people living with HIV, systemic inflammation is high prior to antiretroviral treatment and does not fully normalize following successful viral suppression^45, 46^ leaving them at an increased risk of several inflammation-associated^45, 47^. It is therefore a key observation that marijuana use does not mitigate the inflammation assessed by high plasma CRP levels among those infected with HIV in this cross-sectional sample. An earlier report found decreased CD4 and CD8 T cell activation among heavy marijuana using PLWH who were on antiretroviral treatment, but they also exhibited an increased frequency of classical monocytes, those characterized by high levels of expression of CD14 surface receptors (as well as non-significant decreases in other monocyte subsets)^14^. Given that persistent microbial translocation across the gut epithelium is hypothesized to persist in treated HIV and that classical monocytes robustly respond to microbial products with pro-inflammatory cytokine production^48^, we hypothesize that microbial product-triggered monocyte responses might be greater among heavy marijuana-using PLWH compared to HIV-uninfected heavy marijuana users. Thus, the persistent microbial translocation may be a key difference accounting for lack of decreased CRP associated with heavy marijuana use among the PLWH in this study. Identifying the cause of the lack of response among PLWH in future research is critical to understand how to maximize the potential anti-inflammatory effects of marijuana use among both PLWH and HIV-negative YMSM/TGW.

The use of other substances among this populations appears to have mixed effects on inflammation. Similar to past research observing a significant relationship between the use of various substances and inflammation^20, 21^, our work here observed small-to-moderate correlations between substance use and individual cytokines. Of particular note, we observed several significant correlations between self-reported psychedelic use in the past six months and biomarkers of inflammation but only among PLWH. These findings are in contrast with past work among animal models suggesting the use of psychedelics may have strong anti-inflammatory effects^49, 50^. However, these correlations should be treated only as hypothesis generating and our findings ought to be considered targets for replication in light of the number of tests performed. Future work should aim to further examine the validity of, and mechanism for these findings.

While we found important novel associations between marijuana use and inflammation among our sample, our findings should be considered in the context of their limitations. First, although we believe our study to be the first to assess this relationship among YMSM/TGW, several of the earlier studies did not report sexual and gender minority status; thus, these earlier studies may in fact include YMSM/TGW. Second, we did not perform adjustments for multiple testing when performing the series of Pearson’s correlations. However, Supplemental Tables S1 and S2 provide p-values for such calculations. Future studies should strive to replicate these findings as presented here. Third, we lack data on hepatitis B virus (HBV), hepatitis C virus (HCV), and cytomegalovirus (CMV), as well as other substances which have been shown to affect inflammation (e.g., caffeine), each of which may confound the results observed here. Finally, we obtained only a single CRP measure per participant and thus were unable to account for within-person diurnal variation.

Even in light of these limitations, we observed meaningful findings among this high-risk, substance using sample of YMSM/TGW. First, we observed that the relationship between marijuana and systemic inflammation measured by plasma CRP differed based on HIV status with this relationship being significant only among those uninfected with HIV. Second, we observed no associations between frequency of use of other substances and this measure of systemic inflammation. Further research is needed to assess longitudinal within-person changes in drug use and inflammation among this population, particularly as they have been shown to potentially be at high risk of chronic diseases from an early age.

## Supplementary Information

Supplementary Information
