# Supplementary material for: Plasma C-reactive protein is lower among marijuana using HIV-negative individuals but not among persons living with HIV
Source: Sci Rep. 2021 Mar 1;11:4816. doi: 10.1038/s41598-021-84352-0 (PMC7921677; doi:10.1038/s41598-021-84352-0)
Supplement: Supplementary file 1 — Supplementary Information [file 41598_2021_84352_MOESM1_ESM.docx]

Plasma C-Reactive Protein is Lower Among Marijuana Using HIV-Negative Individuals but Not Among Persons Living with HIV

Ethan Morgan, PhD^1^

Hannah Hudson^2^

Richard D’Aquila^2^

Brian Mustanski, PhD^3,4^

^1^Infectious Disease Institute and College of Nursing, The Ohio State University, Columbus, OH

^2^Division of Infectious Diseases, Department of Medicine, Feinberg School of Medicine, Northwestern University, Chicago, IL

^3^Institute for Sexual and Gender Minority Health and Wellbeing, Northwestern University, Chicago, IL

^4^Department of Medical Social Sciences, Northwestern University, Chicago, IL

**Corresponding Author:**

Ethan Morgan, PhD

1595 Neil Ave, Room 393

Columbus, OH 43210

morgan.1691@osu.edu

**Supplemental Tables**

| **Table S1.** P-values of correlations between inflammatory cytokines, CRP, and various licit and illicit substances among HIV-positive participants, RADAR, Chicago, 2015-2017 | | | | | | | | | |  |
| --- | --- | --- | --- | --- | --- | --- | --- | --- | --- | --- |
| **Variable** | **CRP** | **IL-15** | **MIP-1a** | **MIP-1b** | **IFN-γ** | **IL-10** | **IL-1β** | **IL-6** | **TNF-α** | |
| **BMI** | 0.059 | 0.436 | 0.231 | 0.726 | 0.979 | 0.510 | 0.748 | 0.786 | 0.894 | |
| **Tobacco Use^1^** | 0.357 | 0.926 | 0.646 | 0.895 | 0.471 | 0.420 | 0.230 | 0.529 | 0.947 | |
| **Self-Reported Substance Use^2^** |  |  |  |  |  |  |  |  |  | |
| Marijuana | 0.778 | 0.380 | 0.561 | 0.599 | 0.814 | 0.806 | 0.842 | 0.125 | 0.570 | |
| Poppers | 0.839 | 0.258 | 0.258 | 0.943 | 0.192 | 0.404 | 0.857 | 0.998 | 0.636 | |
| Cocaine/Crack | 0.557 | 0.245 | 0.995 | 0.689 | 0.369 | 0.350 | 0.153 | 0.762 | 0.527 | |
| Ecstasy | 0.573 | 0.732 | 0.307 | 0.202 | 0.726 | 0.752 | 0.428 | 0.680 | 0.111 | |
| Psychedelics | 0.002 | 0.465 | 0.975 | <0.001 | 0.794 | <0.001 | 0.857 | 0.136 | 0.036 | |
| Other Drug Use^3^ | 0.797 | 0.764 | 0.402 | 0.644 | 0.991 | 0.410 | 0.075 | 0.653 | 0.765 | |
| **Urine Drug Screen Positive^4^** |  |  |  |  |  |  |  |  |  | |
| Marijuana | 0.570 | 0.107 | 0.735 | 0.607 | 0.728 | 0.352 | 0.126 | 0.693 | 0.699 | |
| Cocaine/Crack | 0.709 | 0.768 | 0.948 | 0.670 | 0.826 | 0.342 | 0.869 | 0.077 | 0.011 | |
| Ecstasy/MDMA | 0.071 | 0.163 | 0.672 | 0.044 | 0.377 | 0.021 | 0.122 | 0.387 | 0.503 | |
| Other Drug Use^5^ | 0.379 | 0.247 | 0.324 | 0.792 | 0.784 | 0.516 | 0.566 | 0.612 | 0.020 | |
| **AUDIT Score^6^** | 0.454 | 0.873 | 0.456 | 0.405 | 0.590 | 0.613 | 0.821 | 0.825 | 0.587 | |
| **CUDIT Score^6^** | 0.676 | 0.981 | 0.976 | 0.546 | 0.431 | 0.829 | 0.011 | 0.247 | 0.570 | |
| **Rectal gonorrhea** | 0.303 | 0.370 | 0.730 | 0.347 | 0.972 | 0.006 | 0.491 | 0.200 | 0.002 | |
| **Rectal chlamydia** | 0.168 | 0.288 | 0.054 | 0.825 | 0.098 | 0.101 | 0.698 | 0.026 | 0.007 | |
| **Undetectable HIV viral load** | 0.756 | 0.489 | 0.714 | 0.405 | 0.017 | 0.096 | 0.404 | 0.185 | 0.084 | |
| **CRP** | — | 0.267 | 0.205 | <0.001 | <0.001 | <0.001 | 0.100 | <0.001 | <0.001 | |
| ^1^In the past 30 days | | | | | | | | | |  |
| ^2^In the past six months, only those self-reported use ≥4% | | | | | | | | | |  |
| ^3^Substances with <4% self-reported use in the sample: methamphetamines, synthetic marijuana, ketamine, GHB, inhalants, heroin | | | | | | | | | |  |
| ^4^Note: urine drug screens unavailable for poppers and psychedelics | | | | | | | | | |  |
| ^5^Other urine drug screens include: benzodiazepines, amphetamines, methamphetamines, opiates | | | | | | | | | |  |
| ^6^Based on the Alcohol Use Disorders Identification Test (AUDIT) and Cannabis Use Disorders Identification Test (CUDIT) scoring methods; higher score indicates higher risk alcohol use | | | | | | | | | |  |

| **Table S2.** P-values of correlations between inflammatory cytokines, CRP, and various licit and illicit substances among HIV-negative participants, RADAR, Chicago, 2015-2017 | | | | | | | | | |  |
| --- | --- | --- | --- | --- | --- | --- | --- | --- | --- | --- |
| **Variable** | **CRP** | **IL-15** | **MIP-1a** | **MIP-1b** | **IFN-γ** | **IL-10** | **IL-1β** | **IL-6** | **TNF-α** | |
| **BMI** | <0.001 | 0.563 | 0.861 | <0.001 | 0.088 | 0.493 | 0.233 | <0.001 | 0.011 | |
| **Tobacco Use^1^** | 0.240 | 0.462 | 0.810 | 0.840 | 0.700 | 0.061 | 0.791 | 0.570 | 0.293 | |
| **Self-Reported Substance Use^2^** |  |  |  |  |  |  |  |  |  | |
| Marijuana | 0.003 | 0.671 | 0.978 | 0.636 | 0.027 | 0.939 | 0.398 | 0.004 | 0.747 | |
| Poppers | 0.807 | 0.177 | 0.867 | 0.825 | 0.840 | 0.901 | 0.190 | 0.680 | 0.149 | |
| Cocaine/Crack | 0.848 | 0.787 | 0.026 | 0.307 | 0.706 | 0.587 | 0.983 | 0.300 | 0.016 | |
| Ecstasy | 0.785 | 0.316 | 0.758 | 0.362 | 0.477 | 0.674 | 0.591 | 0.301 | 0.421 | |
| Psychedelics | 0.251 | 0.720 | 0.455 | 0.945 | 0.562 | 0.710 | 0.537 | 0.754 | 0.057 | |
| Other Drug Use^3^ | 0.584 | 0.177 | 0.899 | 0.289 | 0.127 | 0.009 | 0.420 | 0.712 | 0.006 | |
| **Urine Drug Screen Positive^4^** |  |  |  |  |  |  |  |  |  | |
| Marijuana | 0.100 | 0.003 | 0.190 | 0.016 | 0.529 | 0.047 | 0.304 | 0.936 | 0.841 | |
| Cocaine/Crack | 0.630 | 0.995 | 0.666 | 0.665 | 0.617 | 0.246 | 0.024 | 0.625 | 0.095 | |
| Ecstasy/MDMA | 0.756 | 0.467 | <0.001 | 0.324 | 0.851 | 0.636 | 0.599 | 0.837 | 0.701 | |
| Other Drug Use^5^ | 0.319 | 0.554 | 0.903 | 0.660 | 0.696 | 0.034 | 0.095 | 0.775 | 0.677 | |
| **AUDIT Score^6^** | 0.104 | 0.012 | 0.541 | 0.216 | 0.210 | 0.894 | <0.001 | 0.215 | 0.245 | |
| **CUDIT Score^6^** | 0.013 | 0.933 | 0.680 | 0.057 | 0.259 | 0.006 | 0.659 | 0.093 | 0.123 | |
| **Rectal gonorrhea** | 0.925 | 0.286 | 0.896 | 0.005 | 0.915 | 0.001 | 0.700 | 0.211 | 0.020 | |
| **Rectal chlamydia** | 0.656 | 0.141 | 0.377 | 0.028 | 0.730 | 0.100 | 0.426 | 0.993 | 0.015 | |
| **Undetectable HIV viral load** | — | — | — | — | — | — | — | — | — | |
| **CRP** | — | 0.003 | 0.749 | 0.022 | <0.001 | <0.001 | 0.773 | <0.001 | <0.001 | |
| ^1^In the past 30 days | | | | | | | | | |  |
| ^2^In the past six months, only those self-reported use ≥4% | | | | | | | | | |  |
| ^3^Substance with <4% self-reported use in the sample: methamphetamines, synthetic marijuana, ketamine, GHB, inhalants, heroin | | | | | | | | | |  |
| ^4^Note: urine drug screens unavailable for poppers and psychedelics | | | | | | | | | |  |
| ^5^Other urine drug screens include: benzodiazepines, amphetamines, methamphetamines, opiates | | | | | | | | | |  |
| ^6^Based on the Alcohol Use Disorders Identification Test (AUDIT) and Cannabis Use Disorders Identification Test (CUDIT) scoring methods; higher score indicates higher risk alcohol use | | | | | | | | | |  |
